# Higher Perceived Stress but Lower Cortisol Levels Found among Young Greek Adults Living in a Stressful Social Environment in Comparison with Swedish Young Adults

**DOI:** 10.1371/journal.pone.0073828

**Published:** 2013-09-16

**Authors:** Åshild Faresjö, Elvar Theodorsson, Marios Chatziarzenis, Vasiliki Sapouna, Hans-Peter Claesson, Jenny Koppner, Tomas Faresjö

**Affiliations:** 1 Division of Community Medicine, Department of Medicine and Health Sciences, Faculty of Health Sciences, Linköping University, Linköping, Sweden; 2 Division of Clinical Chemistry, Department of Clinical and Experimental Medicine, Faculty of Health Sciences, Linköping University, Linköping, Sweden; 3 Elefsina Health Center, Thriasson General Hospital of Elefsina, Athens, Greece; 4 Department of Psychology, School of Philosophy, University of Athens, Athens, Greece; Hunter College, City University of New York (CUNY), CUNY School of Public Health, United States of America

## Abstract

The worldwide financial crisis during recent years has raised concerns of negative public health effects. This is notably evident in southern Europe. In Greece, where the financial austerity has been especially pronounced, the prevalence of mental health problems including depression and suicide has increased, and outbreaks of infectious diseases have risen. The main objective in this study was to investigate whether different indicators of health and stress levels measured by a new biomarker based on cortisol in human hair were different amongst comparable Greek and Swedish young adults, considering that Sweden has been much less affected by the recent economic crises. In this cross-sectional comparative study, young adults from the city of Athens in Greece (n = 124) and from the city of Linkoping in Sweden (n = 112) participated. The data collection comprised answering a questionnaire with different health indicators and hair samples being analyzed for the stress hormone cortisol, a biomarker with the ability to retrospectively measure long-term cortisol exposure. The Greek young adults reported significantly higher perceived stress (p<0.0001), had experienced more serious life events (p = 0.002), had lower hope for the future (p<0.0001), and had significantly more widespread symptoms of depression (p<0.0001) and anxiety (p<0.0001) than the Swedes. But, the Greeks were found to have significantly lower cortisol levels (p<0.0001) than the Swedes, and this difference was still significant in a multivariate regression (p<0.0001), after adjustments for potential intervening variables. A variety of factors related to differences in the physical or socio-cultural environment between the two sites, might possibly explain this finding. However, a potential biological mechanism is that long-term stress exposure could lead to a lowering of the cortisol levels. This study points out a possible hypothesis that the cortisol levels of the Greek young adults might have been suppressed and their HPA-axis down-regulated after living in a stressful environment with economic and social pressure.

## Introduction

The financial crisis in Europe during recent years has raised concerns of how this will affect the health of people especially in Greece and southern Europe [1]. This crisis and changes in the economy – particularly it's effect on unemployment – is stressful and will adversely affect population health [2]. Stress-related disorders constitute an increasing public health problem globally, and the WHO has declared that along with mental health problems, stress-related disorders are major causes of early death in Europe [3]. Recession in the national economy leading to high unemployment rates has been shown to correlate with decreased quality of life, physical and mental illness such as anxiety, depression, and climbing suicide rates, and increased levels of the stress hormone cortisol [4], [5]. This has been suggested to be the result of increased negative mental conditions such as insecurity for the future, rising demands of adaptation, and loss of protective social networks [6]. It is important to note, however, that individuals react differently and can be more or less vulnerable to stressors. Economic crises are a type of community stressor that could affect a country and its whole population in many different ways, such as destabilization in the labor market, increased unemployment rates, and reductions in the public sector and the social security systems [2]. After some years of economic growth, Greece was hit by the financial crisis starting with an economic recession from 2008. The health impacts in recent years of the economic crisis in Greece has been connected to diminishing perceived health and quality of life, increased prevalence rates of mental health problems including an increased risk for depression by 2.6 times in 2011 compared to 2008, and a rise in the suicide rate of almost 20% in the Greek population as the crisis has developed [7], [8], [9].

The steroid hormone cortisol plays a crucial role in the stress response, and is increased in situations perceived as stressful to the organism. Measuring the concentration of cortisol in blood, saliva, and urine are established methods for momentary assessments of the activity in the hypothalamic-pituitary-adrenocortical axis (HPA) – for example salivary cortisol values only relates to the previous 20 minutes [10]. Some of the functions of cortisol in the body are to recruit energy from adipose and muscular tissues and to suppress the immune system. If the cortisol levels become too high or too low for a longer period, a state of hyper- or hypocortisolism is present, and both are associated with stress-related disease [11]. Hypercortisolism is associated with a number of various diseases, e.g., cardiovascular diseases, type-2 diabetes, depression, and slow wound-healing [12], [13], [14], [15], [16].

Until recently, it has only been possible to measure physiological stress by analyzing cortisol in blood, saliva, or urine samples. The shortcoming of these methods is that they cannot detect stress longitudinally since they only indicate stress over a short time interval. Further, individual cortisol levels can also fluctuate depending on a wide array of factors and be influenced by the situation as well as the time of day due to the circadian rhythm [17, 18), food intake [19], and also exercise habits [20]. The new method measuring cortisol in hair has been developed with the ability to retrospectively measure the mean cortisol levels over time, which diminishes these shortcomings, and makes it possible to measure long-term cortisol exposure [21], [22]. Cortisol in hair merely specifies cortisol levels as the cumulative activity of the HPA axis, although it is not known whether this is due to one stressful event per se, or numerous acute stress experiences since it is a mean value over a time period. Research indicates that hair can be used as a retrospective calendar for months, and the evidence is growing for using cortisol in hair as a new biomarker of systemic stress both from animal and human studies [23], [24]. There is also research exploring the possible connection between disease or adverse social environment and elevated levels of cortisol in hair, and such connections have been found with myocardial infarction, chronic pain, and long-term unemployment [25], [26], [27].

Growing evidence has emerged of the relationship between stress and hypocortisolism [11], [28]. Pathological conditions in which this association has been shown relate to, for example, post-traumatic stress disorder (PTSD), chronic fatigue syndrome, autoimmune disease, and fibromyalgia [11], [14]. The mechanisms of stress-induced hypocortisolism are not clear, but may reflect many aspects of neuroendocrine dysregulation, which may differ within and across populations, leading to a lack of cortisol effects in the organism [28]. Animal studies of macaques has shown a probable mechanism in that after a long period of stress with excessive cortisol release and a hyperactive HPA-axis, this becomes fatigued and can no longer uphold even a normal cortisol level [29]. Another important factor could be a general feeling of worry and anxiety in the population that could prolong the stress exposure and keep it at a high level even if people are not objectively exposed to the stressors [30].

The objective of this study was to investigate whether different health indicators and stress levels measured by cortisol concentrations in extracts of hair were different amongst comparable Greek and Swedish young adults, considering the fact that Greece has been especially affected by the recent economic and social crisis, whereas Sweden has been much less affected.

## Methods

### Participants

This is a cross-sectional study among young adults from Athens in Greece and the city of Linköping in south-eastern Sweden. The participants were all university students recruited from Athens University and Linköping University, studying in their second or third year in the medical or psychology programs. The data collection comprised answering a questionnaire and taking hair samples. The total number of participants in the study was n = 114 Swedish and n = 125 Greek students, and the participation rate was 66% in Sweden and 63% in Greece. Important to note was the exclusion of students with hair shorter than 3 cm, since this was an exclusion criteria. These “natural drop-outs” were mainly men on both sites. The final numbers of included participants were, however, slightly diminished: two Swedish and one Greek participant were omitted from further analysis since they were considered as statistical outliers in the cortisol analysis. This resulted in a final sample of n = 112 Swedish (n = 69 women and n = 43 males) and n = 124 Greek (n = 115 women and n = 9 males) participants.

All participants gave their written informed consent to participate in the study before the collection of hair samples was done. The Research Ethics Committee at the Faculty of Health Sciences, Linköping University, Sweden and The Research Ethics Committee at Athens University, Greece approved the study in 2012. All original research data like questionnaires etc. in this study are stored for at least 10 years and are available upon requests to the researchers.

### Procedures and Measures

A questionnaire including validated and previously tested questions [21] was used measuring sociodemographic variables including: age, sex, and self-reports of longstanding chronic illness (coronary heart disease, diabetes, cancers, or rheumatic disorders), and potential intervening factors like smoking. Possible confounders within the previous 3 months included: permed or colored hair (it was not specified in the questionnaire if the hair was permed or colored), regular medication in general and regular medication of glucocorticoids like steroid creams, nose sprays, or inhalation aerosols (no specification was made in the questionnaire which specific type of these glucocorticoids the respondent used). Further, experiences of serious life-events during the last three months such as divorce, unemployment, surgery, economical problems, serious illness, or a death in family were recorded [31]. Self-reported health was measured by three categories: not so good, average, and good. The variable “hope for the future” was measured in five categories: completely hopeless, hopeless, neither hopeless or hopeful, partially hopeful, or very hopeful. The variable “hope for the future” was for illustrative purposes divided into three groups in table 1. Included in the questionnaire were also The Hospital Anxiety and Depression Scale (HAD) and the Perceived Self-rated Stress Scale (PSS 10-itemversion) [32]. Swedish and Greek translations were used for both PSS and HAD. The scales HAD depression and HAD anxiety were respectively divided into three groups and illustrated in table 1, following the established clinical cut-offs [33]. PSS was for illustrative purposes in table 1, divided into three groups (0–10p, 11–20p, 21–40p), as it has no established clinical cut-offs.

**Table 1:** Characteristics of different variables for the Greek and Swedish sample.

Different variables		Greek (n = 124) n (%)	Swedish (n = 112) n (%)	p-value (correlation)
Daily smoker	No	109 (88%)	110 (98%)	0.002
	Yes	15 (12%)	2 (2%)	
Colored or permed hair	No	88 (71%)	86 (77%)	0.30
	Yes	36 (29%)	25 (23%)	
Experience of serious life events	No	71 (58%)	86 (77%)	0.002
	Yes	52 (42%)	26 (23%)	
Regular medication	No	104 (84%)	74 (66%)	0.002
	Yes	20 (16%)	38 (34%)	
Medication with glucocorticoids	No	115 (93%)	99 (88%)	0.27
	Yes	9 (7%)	13 (12%)	
Longstanding illness	No	112 (90%)	96 (86%)	0.32
	Yes	12 (10%)	16 (14%)	
Self-reported health	Not so good	7 (6%)	7 (6%)	0.89
	Average	12 (10%)	13 (12%)	
	Good	103 (84%)	92 (82%)	
Hope for the future	Hopeless	24 (20%)	5 (5%)	<0.0001(−0.385)
	Neither	34 (27%)	15 (13%)	
	Hopeful	65 (53%)	92 (82%)	
Perceived stress (PSS)	Not stressed (0–10)	8 (7%)	32 (29%)	<0.0001 (0.386)
	Moderate (11–20)	57 (46%)	56 (50%)	
	Stressed (21–40)	58 (47%)	24 (21%)	
HAD Depression	No (0–7)	106 (86%)	108 (96%)	<0.0001 (0.271)
	Moderate (8–10)	12 (10%)	3 (3%)	
	Yes (>10)	5 (4%)	1 (1%)	
HAD Anxiety	No (0–7)	55 (45%)	72 (64%)	<0.0001 (0.217)
	Moderate (8–10)	32 (26%)	21 (19%)	
	Yes (>10)	36 (29%)	19 (17%)	

Collection, extraction, and analysis of cortisol in the hair samples followed a procedure previously described [21]. In short, an approximately 3-mm thick piece of hair was cut off close to the scalp from the posterior vertex area of the head. No hair was shorter than 3 cm in length, and all participants donated sufficient hair volume for the analysis. Cortisol was measured based on the established competitive radio immunoassay method [34]. Hair samples were weighed on a Sartorius R 180 D micro-scale and homogenized using a Retch Tissue Lyzer II. Aluminum cylinders were made accommodating four 2-mL tubes containing pre-weighed hair samples and one 0.5-mm steel pellet. These were frozen in liquid nitrogen for 2 minutes, after which the hair samples were homogenized for 2 minutes. Methanol (1 ml) was added to each tube and the samples extracted for 24 hours on a moving board constantly keeping the steel pellets in soft motion within the tubes. 0.8 mL of the methanol supernatant was pipetted off and lyophilized using a Savant Speed Vac Plus SC210A and the samples were dissolved in radioimmunoassay buffer. Hair samples of 3–5 mg were needed for reliable results and maintaining a total inter-assay coefficient of variation below 8% for hair extraction and measurement of cortisol by the radioimmunoassay. In this study, we used 5–6 mg hairs for the analysis. Taking the binding of cortisol as 100%, the antiserum cross-reacts 137% with 5α-dihydroxycortisol, 35.9% with 21-deoxycortisol, and 35.9% with prednisolone, but less than 1% with endogenous steroids.

### Statistical Analysis

**Figure 1:**
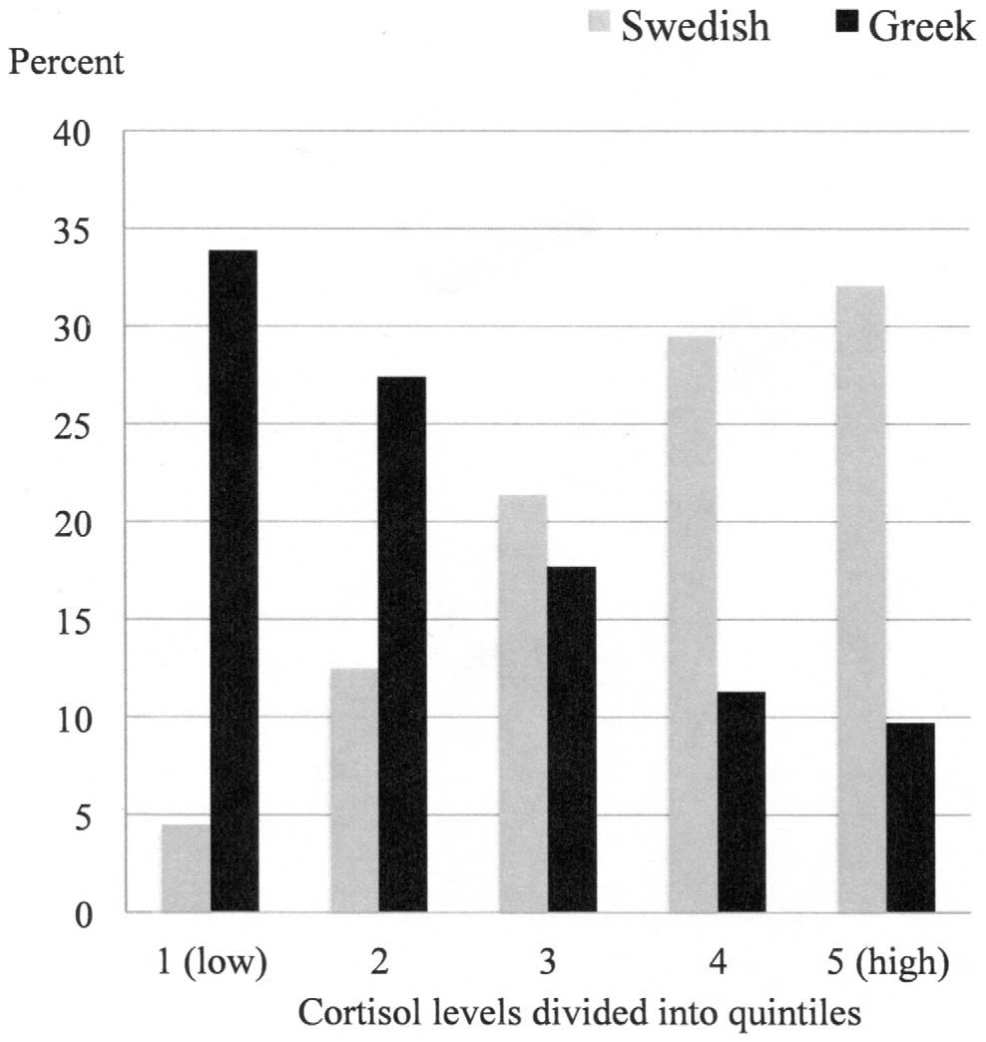
Distribution (percentage) of cortisol levels (pmol/g) divided into quintiles, for Greek (black) and Swedish (grey) young adults; 1. (LOW) 4.3–12.3 pmol/g 2. 12.4–16.4 pmol/g 3. 16.5–22.1 pmol/g 4. 22.2–30.1 pmol/g 5. (HIGH) 30.2–168.0 pmol/g.

For bivariate tests between groups in table 1, the Chi^2^ test and Pearson's correlation was applied. The measured cortisol values were logarithmized prior to the statistical analysis due to skewness in the distribution. However, means presented in table 2 are based on raw values for full clarity. ANOVA and t-test were used for comparison of means in the bivariate tests. For illustrative purposes in the tables, the variables “Hope for the future”, Perceived Stress Scale, HAD Depression, and Had Anxiety were divided into three groups. The cortisol values were divided into quintiles for illustrative purposes in Figure 1. Variables that were statistical significant or close to be significant associated to cortisol levels, were included in the final multivariate linear regression model, but included were also the variables sex and age. A p-value of p<0.05 was considered statistically significant. All statistical analyses were made with the IBM (SPSS) software, version 20.

**Table 2:** Different indicators and their association to cortisol levels.

Different indicators		Cortisol levels pmol/g Mean (SD)		p-value
Swedish sample (n = 112)	No (n = 219)	31.6	22.0	<0.0001
Greek sample (n = 124)	Yes (n = 19)	19.8	21.3	
Daily smoker	No (n = 219)	25.8	23.0	0.34
	Yes (n = 19)	20.4	9.4	
Colored or permed hair	No (n = 174)	24.8	20.4	0.58
	Yes (n = 61)	26.0	27.3	
Experience of serious life events	No (n = 157)	26.0	20.9	0.61
	Yes (n = 78)	24.4	25.2	
Regular medication	No (n = 178)	24.0	18.8	0.10
	Yes (n = 58)	29.7	30.7	
Medication with glucocorticoids	No (n = 214)	24.8	20.9	0.16
	Yes (n = 22)	31.8	33.5	
Longstanding illness	No (n = 208)	24.3	19.8	0.04
	Yes (n = 28)	33.5	35.7	
Self-reported health	Not so good (n = 14)	35.4	32.3	0.02
	Average (n = 25)	31.5	38.3	
	Good (n = 195)	24.1	18.4	
Hope for the future	Hopeless (n = 29)	34.5	39.4	0.06
	Neither (n = 49)	18.3	11.1	
	Hopeful (n = 157)	26.1	20.1	
Perceived stress (PSS)	Not stressed (0–10) (n = 40)	29.4	23.5	0.31
	Moderate (11–20) (n = 113)	24.5	18.9	
	Stressed (21–40) (n = 82)	24.9	26.1	
HAD Depression	No (0–7) (n = 214)	24.5	19.7	0.02
	Moderate (8–10) (n = 15)	27.7	29.3	
	Yes (>10) (n = 6)	48.8	61.1	
HAD Anxiety	No (0–7) (n = 127)	26.5	21.6	0.57
	Moderate (8–10) (n = 53)	22.6	15.7	
	Yes (>10) (n = 55)	25.3	28.7	

## Results

The mean cortisol levels of the total study sample (N = 236) were: 25.4 pmol/g (SD 22.4). The Greek mean cortisol levels were 19.8 pmol/g (SD 21.3), and the Swedish 31.6 pmol/g (SD22.0). Greek cortisol levels were significantly lower (p<0.0001) than comparable Swedish young adults, also after adjustments for differences in sex and age distribution between the sites. In Figure 1, the distribution of cortisol levels divided into quintiles for the Greek and Swedish populations is illustrated.

A difference in sex distribution was evident with a higher excess of females in the Greek sample than in the Swedish sample (p<0.0001), and the mean age of the Swedish sample (23.1 years, SD 3.6) was significantly higher (p = 0.001) than for the Greeks (20.5 years, SD 2.5). Some indicators for the Greek and Swedish samples are shown in table 1. There were no differences between the samples concerning colored or permed hair, or in take of pharmaceuticals containing glucocorticoids, but regular medication was significantly (p = 0.002) more frequent among the Swedes. The number of daily smokers was significantly higher (p = 0.002) among the Greeks compared to the Swedes. The Greek sample reported significantly more experiences of serious life events (p = 0.002), higher perceived stress (PSS) (p<0.0001), higher scores for HAD depression (p<0.0001) and HAD anxiety (p<0.0001), and lower scores for hope for the future (p<0.0001). No differences between the sites were found concerning self-reported health.

The mean cortisol concentration for different indicators is shown in table 2. There were no sex (p = 0.57) or age (p = 0.14) differences in mean cortisol levels. Some of the indicators were significantly associated to cortisol levels like; “longstanding illness” (p = 0.04), “self-reported health” (p = 0.02), and HAD depression (p = 0.02). The variable “hope for the future” almost reached the chosen significance level of p<0.05. The potential confounders – daily smoker (p = 0.34), colored or permed hair (p = 0.58), regular medication (p = 0.10), and medication with synthetic glucocorticoids (p = 0.16), were not statistically significantly associated to cortisol levels.

In table 3, a multivariate analysis, revealed that the difference between the Greek and Swedish sample persisted (p<0.0001) even when adjusted for other potential intervening variables, none of the other variables remained significant in the multivariate analysis.

**Table 3:** Multivariate regression of different independent variables with logarithm cortisol levels as dependent variable.

Independent variables	Standardized Beta coefficient	95% confidence interval for Beta low/high	t-value	p-value
Constant		−4.21/−2.42	−7.268	<0.0001
Sweden – Greece	−0.457	−0.75/−0.37	−5.793	<0.0001
Sex	0.045	−0.13/0.26	0.683	0.50
Age	0.004	−0.24/0.25	0.056	0.96
Hope for the future	0.016	−0.08/0.96	0.188	0.85
Self-reported health	−0.037	−0.13/ 0.07	−0.522	0.60
Longstanding illness	0.057	−0.15/0.36	0.842	0.40
Regular medication	−0.076	−0.32/0.10	−1.007	0.32
Medication with glucocorticoids	0.082	−0.14/0.49	1.073	0.28
HAD Depression	0.060	−0.02/0.04	0.779	0.44

Multivariate linear regression model (with logarithm cortisol values as dependent variable), df = 9, R Square = 0.20, F = 6.29, p<0.0001.

## Discussion

The main finding in this study was that young Greek adults had significantly lower cortisol levels than comparable Swedish young adults, despite that the Greeks reported higher perceived stress, reported more experience of serious life events, had lower hope for the future, and had widespread symptoms of depression and anxiety.

The differences in cortisol levels found in this study could reflect a variety of possible differences between young adults living in these two countries. The Mediterranean climate with extensive sun exposure might over time decrease the concentration of cortisol in hair by a type of leaching, but no such results have yet been reported for cortisol concentration in human hair. The warmer Greek climate might affect the hair growth speed, and, if so, the cortisol concentration would be diluted and therefore lower than for the Swedes. A warm climate has to our knowledge not been confirmed in earlier studies as having any role on hair growth speed. The cortisol levels were not associated with permed or coloured hair, which is in accordance to results from other studies [35]. The influences of natural hair color on cortisol levels have been evaluated and no significant variations between dark or blond hair were seen [36]. Some minor diversity of hair growth profiles has been reported between ethnic populations worldwide [37]. This European study solely constitutes of a Caucasian population, although some genetic differences of potential relevance between Mediterranean and northern European populations cannot be ruled out. Another potential confounder could be differences in the use of medications containing glucocorticoids [38]. In both countries these types of pharmaceuticals are available over the counter and there were no significant differences found in the use of these medications between the sites in this study. Other possible explanations that might have affected the differences in cortisol levels are related to cultural and psychosocial differences like perception of stress, coping styles, and personalities [39], [40] but such potential differences were not further studied in this study and could therefore only be anecdotal.

All health indicators measured in this study point in the same direction: the Greek young adults reported lower health status than Swedish. One could therefore expect that their cortisol levels should be higher since a broad area of research has shown that recent or on-going stress generally seems to be associated with increased hair cortisol levels [41]. However, the young Greeks had on the contrary significantly lower cortisol levels than the Swedes. A hypothesis to explain this phenomenon could be that the cortisol levels of the Greek young adults might have been suppressed after living in an environment with economic and social pressure. Although our results reveal lower cortisol levels in the Greek subjects, we cannot label these as hypocortisolism, which is diagnosed in clinical settings. However, this finding is comparable with other studies where individuals under long-term stress exposure and trauma show a down-regulation of their HPA axis [42]. The basic mechanism of the HPA axis is that stress first leads to hyperactive functioning, but if the stress exposure is longstanding and individuals are no longer able to cope with this exposure, a state of exhaustion is reached and the system turns to hypoactive functioning. This tendency of hypocortisolism has been reported for patients with a variety of stress-related disorders such as chronic fatigue syndrome, fibromyalgia, lower-back pain, post-traumatic stress disorder, and burnout [33], [43], [44], [45].

The results of this study should be considered in light of some limitations. Only young adults – i.e., university students – were included, which might indicate that these groups should be less affected by the economic crisis than the general population. Although these groups are not in the labor force, they do not live their lives isolated from the rest of the community. Therefore, they are at least indirectly affected in their daily life by the economic and social crises as the rest of the Greek population. The cross-sectional study design is another limitation in this study, since we did not measure changes of cortisol levels over time, i.e., before and after the Greek economic crisis. The uneven female-male ratio (in total only 22% were males), especially the low participation of Greek males, is another study limitation. The data collection in both sites yielded randomly selected university classes (medical and psychology students) and by chance the number of males were relatively low in the selected classes in Greece leading to a more uneven female-male ratio. In addition, a general limitation of the cortisol in the hair method itself is that enough hair length of the participants (around 3 cm hair) was needed, which excluded many males. Strengths in this study are the inclusion of both biological markers and indicators of self-reports of stress. Another strength is the comparison between defined and comparable groups in two countries, and the focus could instead be on their health situations in their respective social environments. The measurement of cortisol in hair has been studied for several years and has great potential as a biomarker measuring prolonged cortisol exposure [41].

The down-regulation of the HPA axis is a mechanism that biologically copes with the long-term exposure to a stressful social environment. A reduced HPA axis reactivity in chronically stressed individuals is maladaptive since it is also linked to the immune system response. The financial crisis in southern Europe has posed major threats to public health, where not only suicides, but also new outbreaks of infectious diseases are becoming more common [1]. To be repeatedly exposed to intense stimuli of a high allostatic load could lead to a lowering of the cortisol levels, and possibly also a reduced immune defense with harmful health effects in humans [11], [46].

Living everyday in a social environment affected by the economic and social crisis with high unemployment rates, reduced salaries, and reduction of the social security nets that Greece has for some years experienced, is stressful for the whole population [7], [8]. Although the coping strategies to handle this type of stressful situation could vary in the population, there also might be social or cultural differences in this respect. General worries and feelings of anxiety in the population could keep the stress exposure at high levels for a long period of time, even if the immediate threat has been reduced [30]. People are directly or indirectly affected, and the young adults followed in this study are no exception, and even though they are not active in working life, worries of their life situation and concerns for their family and future arises.

### Conclusions

Young Greek adults living in an environment under economic and social strain reported significantly higher perceived stress, more frequent depression and anxiety, and lower hope for the future than comparable Swedish young adults not exposed to this crisis. The Greeks were found to have significantly lower levels of the stress hormone cortisol, measured in hair, than the Swedish young adults. These differences in cortisol levels might be seen against the background of differences in physical and socio-cultural environment between these two countries. However, besides these explanations, this study also raises a hypothesis of a possible biological mechanism that the Greeks' cortisol levels might have been suppressed and their HPA-axis down-regulated after living in a stressful economic and social environment. More studies are warranted to follow how economic and social crises may affect the biomedical stress-response mechanism and disease risks in a longer perspective.
